# Sequential release of cytokines, lipid mediators and nitric oxide in experimental colitis

**DOI:** 10.1155/S0962935195000305

**Published:** 1995-05

**Authors:** A. P. M. van Dijk, Z. J. Keuskamp, J. H. P. Wilson, F. J. Zijlstra

**Affiliations:** 1 Department of Pharmacology Erasmus University Rotterdam The Netherlands; 2 Department of Internal Medicine II University Hospital Dijkzigt Rotterdam The Netherlands

## Abstract

The object of this study was to establish whether different pro- and anti-inflammatory mediators were formed in colonic tissue from experimental colitis depending on the course of the disease. Concentrations of mediators of inflammation were examined in colonic tissue in dextran induced colitis in mice. Initial inflammation was produced by 5 days treatment of 10% dextran sodium sulfate (DSS) in drinking water, followed by a further 9 day period of 2% DSS in an attempt to produce a milder chronic inflammation. The degree of inflammation was scored by a standardized macroscopic and histological examination. Initially, a 60% maximum inflammation score was observed at day 4. At this time inflammation was associated with the release of interleukin-lβ (IL-1β) and tumour necrosis factor-α (TNFα), whereas both prostaglandins 6kPGF_1α_ and PGE_2_ and nitric oxide (NO) markedly decreased. Then a 25% inflammation score was reached which coincided with an increased production of platelet-activating factor (PAF). No significant changes were observed in leukotriene B_4_ and C_4_ formation. In conclusion, pro-inflammatory cytokines IL-1β and TNFα are considered to be primary mediators, whereas PAF, eicosanoids and NO may reflect secondary mediators in experimental colitis.


Mediators of Inflammation 4, 186–190 (1995)

### Sequential release of cytokines, lipid mediators and nitric oxide in experimental colitis

A. P. M. van Dijk,[1,CA] Z. J. Keuskamp,[1] J. H. P. Wilson[2] and F. J. Zijlstra[1]

[1]Department of Pharmacology, Erasmus University, Rotterdam; and
[2]Department of Internal Medicine II, University Hospital Dijkzigt, Rotterdam, the Netherlands

[CA]Corresponding Author

**The object of this study was to establish whether different pro- and anti-inflammatory mediators were formed in colonic tissue from experimental colitis depending on the course of the disease. Concentrations of mediators of inflammation were examined in colonic tissue in dextran induced colitis in mice. Initial inflammation was produced by 5 days treatment of 10% dextran sodium sulfate (DSS) in drinking water, followed by a further 9 day period of 2% DSS in an attempt to produce a milder chronic inflammation. The degree of inflammation was scored by a standardized macroscopic and histological examination. Initially, a 60% maximum inflammation score was observed at day 4. At this time inflammation was associated with the release of interleukin-1β (IL-1β) and tumour necrosis factor-α (TNFα), whereas both prostaglandins $6kPGF_{1\alpha}$ and $PGE_2$ and nitric oxide (NO) markedly decreased. Then a 25% inflammation score was reached which coincided with an increased production of platelet-activating factor (PAF). No significant changes were observed in leukotriene $B_4$ and $C_4$ formation. In conclusion, pro-inflammatory cytokines IL-1β and TNFα are considered to be primary mediators, whereas PAF, eicosanoids and NO may reflect secondary mediators in experimental colitis.**

**Key words:** Dextran colitis in mice, Eicosanoids, IL-1β, NO, PAF, TNFα

### Introduction

Several experimental models have been described which resemble human inflammatory bowel disease (IBD), including ulcerative colitis (UC) and Crohn's disease (CD).[1] These animal models have been used to examine the production of inflammatory mediators by intestinal mucosa and to determine the effects of various drugs on inflammation and on individual mediators.[2,3] The customary drugs used in IBD—corticosteroids and 5-aminosalicylates—are thought to act by interfering with eicosanoid production.[4] In IBD, the synthesis of leukotriene $B_4$ ($LTB_4$) in colonic mucosa has been shown to be enhanced compared with its production in normal tissue.[5] However, specific 5-lipoxygenase inhibitors such as Zileuton have not been shown to have marked beneficial effects on inflammation in man, although $LTB_4$ levels in dialysates were decreased.[6] Prostaglandin $E_2$ ($PGE_2$) is also increased in dialysates[7] and mucosal tissue[8] of patients with UC and CD. However, the anti-inflammatory effects of this substance are well documented,[9] and it is therefore likely that $PGE_2$ mainly acts as a feed-back bioregulator of eicosanoid and cytokine production.[10] Since damage of the endothelial cell can be a prime event leading to mucosal injury, this could reflect a diminished release of the endothelial derived mediators prostacyclin ($PGI_2$) and nitric oxide (NO). On the contrary, we have found high amounts of the 15-lipoxygenase product 15-hydroxy-eicosatetraenoic acid (15-HETE) in human colonic mucosa.[11] This substance is synthesized both by endothelial cells and macrophages. The minor anti-inflammatory effects of 15-H(P)ETE could be ascribed to inhibition of the pro-inflammatory mediator $LTB_4$.[12] It has been suggested that cytokines such as tumour necrosis factor (TNFα) and interleukin-I (IL-1) are primary triggers of the initial inflammation,[13] whereas platelet-activating factor (PAF) is considered to be involved as a secondary trigger, resulting in a chronic phase of the inflammation.[14] This hypothesis implies that the choice of drug therapy for IBD might depend on the stage of the disease. In the search for a suitable model for UC resembling acute and chronic inflammation of colonic mucosa, we modified the dextran sodium sulfate (DSS) induced colitis in mice and measured a

© 1995 Rapid Communications of Oxford Ltd wide range of mediators of inflammation during the course of the disease.

### Materials and Methods

*Induction of colitis:* Colitis was induced in BALB/c female mice (20–22 g) by adding high-molecular dextran sodium sulfate (DSS; Pharmacia, Sweden) to their drinking water, which was given to the mice *ad libitum* from the first day of the treatment. DSS, given orally, causes a diffuse inflammation of the mucosa of the entire gut. The mechanism by which this inflammation is induced is not entirely clear, but bacterial flora in the gut appear necessary for the process.[15] During the treatment schedule, the concentration of DSS in the water was altered. For the first 5 days 10% (w/v) DSS was administered via the drinking water in an attempt to provide a model for the acute and initial phase of inflammation. During the last 9 days of the schedule, the concentration was lowered to 2% (w/v) DSS, in order to create a more chronic and milder inflammation in the mucosa of the colon.

*Treatment schedule:* In this study 29 mice were randomly divided into five groups. The first group consisted of nine mice and served as a control group throughout the investigation period. The other 20 mice were divided into four groups of five mice each. The animals were killed by cervical dislocation after 2, 4, 8 and 14 days.

*Experimental procedure and inflammation score:* After the mice were killed, the colon from flexure to rectum was removed immediately. A small piece was cut off and stored in formaldehyde, and subsequently prepared for histological examination. The colon was opened in a transverse direction and examined macroscopically using a 10× magnification. Signs of inflammation and changes in the appearance of the colonic mucosa were evaluated and the severity of inflammation (erythema, haemorrhages, mucus, oedema, strictures, diarrhoea) was scored (0–6). Faeces were also examined for the presence of occult blood (score 0–1). After macroscopic assessment, the colon was incubated in 5 ml 0.9% w/v saline and then shaken lightly for 5 min. This suspension was centrifuged (2800 × g, 10 min, 4°C) and the supernatant was stored at –80°C. The colon was weighed and 2 ml KREBS buffer added. The colonic tissue was fragmented in this solution using the Ultra-Turrax (Polytron, Switzerland), after which this suspension was centrifuged (10 000 × g, 10 min, 4°C) and the supernatant stored at –80°C until measurements of inflammatory mediators could be done at the same time in one assay.

Microscope evaluation of the colonic tissue was assessed as follows: cell infiltration of the tissue; depletion of goblet cells; loss of the tissue architecture; and the formation of crypt abscesses (score 0–4). Each of the given parameters on macroscopic and microscopic evaluation was subdivided into a three-scaled point system (+, ++ and +++), resulting in a total inflammation score range of 0–33 points, which served as an objective measure of the severity of inflammation of the colonic tissue.[12]

*Measurement of mediators:* The amount (mg) of protein per g wet homogenized tissue was determined in the supernatant by a micro-scale method (Instruchemie, Hilversum, The Netherlands) using an ELISA reader (Biorad, USA) at 600 nm. Myeloperoxidase (MPO) in these samples was determined by means of a double antibody radioimmunoassay (RIA, Pharmacia, Sweden) in which bound and free MPO were separated by addition of a second antibody immunosorbent and $^{125}$I was counted.

The concentrations of different mediators of inflammation were measured in duplicate in the liquid samples described above. The nitric oxide metabolites, nitrate (after reduction to nitrite) + nitrite, were measured colometrically after the Griess reaction, as described by Phizackerly and Al-Dabbagh.[16] Briefly, the following modifications were applied:[17] samples were deproteinized by adding 1M NaOH and 1.3 M $ZnSO_4$; nitrate was converted to nitrite by the addition of *Klebsiella*-pneumonia suspension, 0.2 M *N*-tris-(hydroxymethyl)-methyl-2-amino-ethane sulfonic acid (TES) and 0.5 M sodium formate; after anaerobic incubation, water was added and nitrite was assayed in supernatants. Nitrite was estimated in deproteinized samples, mixed with 1% sulfanilamide in 15% phosphoric acid; after the addition of 0.1% *N*-(1-naphthyl)ethylenediamine, the absorption was determined at 540 nm by means of an ELISA reader (Biorad). The linear detection range was between 1 and 250 μM.

Cytokines, eicosanoids and PAF were determined in the samples of the colonic tissue by means of ELISAs and RIAs. For the assay of TNEα, a specific mouse TNFα ELISA kit was obtained from Genzyme Corp., using a hamster monoclonal antibody specific for murine TNF, a goat polyclonal anti-murine TNF antibody, and, as third antibody, horsedish peroxidase-conjugated donkey anti-goat Ig. The peroxidase enzyme reacted with peroxide substrate, after which the colour intensity was measured at 492 nm. Detection range was between 0.05 and 1.6 ng/ml. Measurements of IL-1β were performed

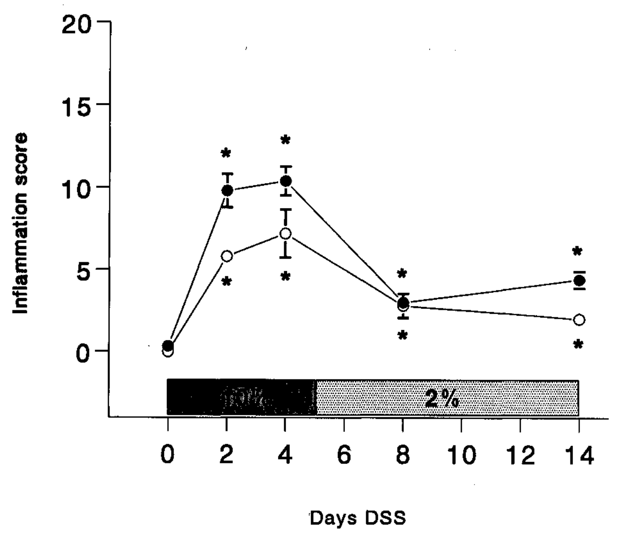

FIG. 1. Macroscopic (●) and microscopic (○) inflammation scores of mouse colonic mucosa (means ± S.E.M.) after dextran sodium sulfate application. *$p < 0.05$ *vs.* day 0.

using a high sensitivity IL-1β assay from Cistron Biotechnology (Eurogenetics, Belgium), bound to a solid phase monoclonal antibody, after which in the second stage polyclonal rabbit anti-IL-1β was added, followed by goat anti-rabbit IgG conjugated to horseradish peroxidase enzyme which reacts with peroxide substrate. The end product was measured at 450 nm. Detection range was between 4 and 250 pg/ml.

Eicosanoids were determined by RIA. Specific antibodies were obtained from Advanced Magnetics Inc. (MA, USA), tritiated compounds from Amersham (UK) and standards from Sigma Co. (USA). Assays were performed for a 16 h competitive binding period, using standard curves from 0–500 pg/tube; sensitivities were: $PGE_2$, 2 pg/tube; $6kPGF_{1\alpha}$, $LTB_4$ and $LTC_4$, 5 pg/tube at 98% $B/B_0$.

Finally, PAF was measured using a SPA–RIA kit assay system of Amersham (UK). The standard curve was prepared from 0 to 1280 pg/tube and a sensitivity of 20 pg/tube. The concentrations of the mediators produced during inflammation were expressed per mg wet colonic tissue.

*Statistics:* Data are expressed as the mean ± standard error of the mean (S.E.M.). Data were analysed statistically with ANOVA followed by Student's *t*-test and *p* values $< 0.05$ (two-sided) were considered significant.

### Results

*Inflammation score:* The results of the macroscopic and microscopic inflammation scores are

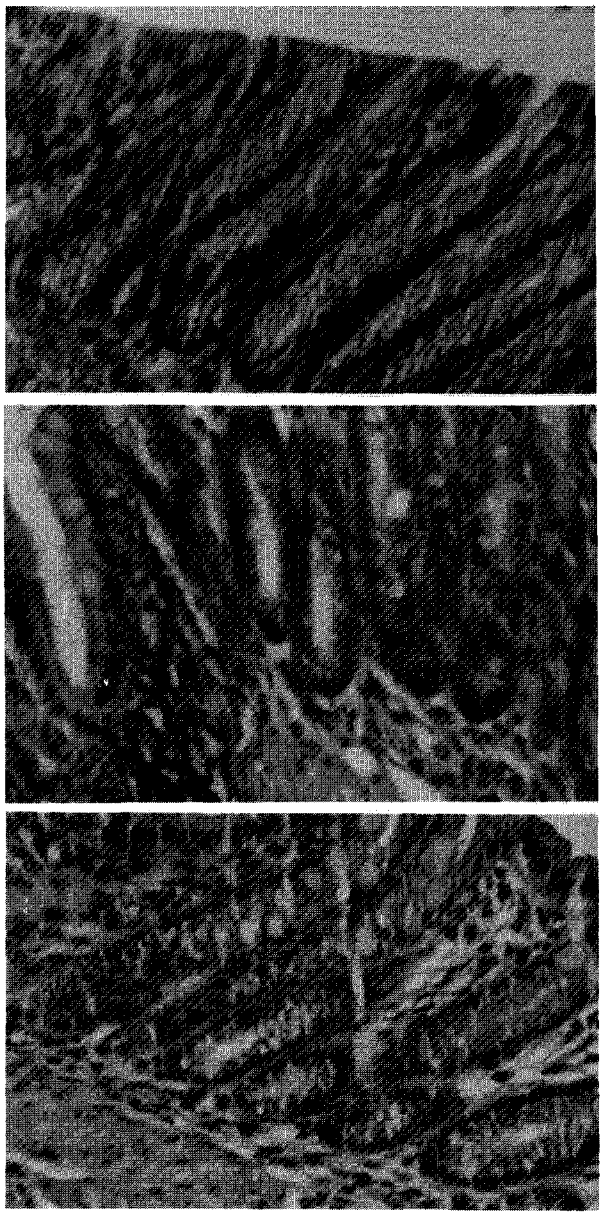

FIG. 2. Histological sections of rectal colonic mucosa; (a) section from a control mouse in which no histological damage was observed; (b) section of rectum after 4 days treatment with DSS showing cell infiltration, goblet cell depletion and loss of architecture; (c) section of rectum after 8 days treatment with DSS showing cell-less infiltration and some goblet cell depletion.

shown in Fig. 1. In the control group, which contained mice that had not been treated with DSS, no inflammatory activity was observed (Fig. 2(a)).

The group treated for 2 days with 10% DSS solution and then killed at the second day of the treatment schedule, showed a significant increase in the inflammation score. The maximum inflammation score was seen after 4 days treatment with 10% DSS (Fig. 2(b)). The inflammation score of the fourth and fifth group, in which the

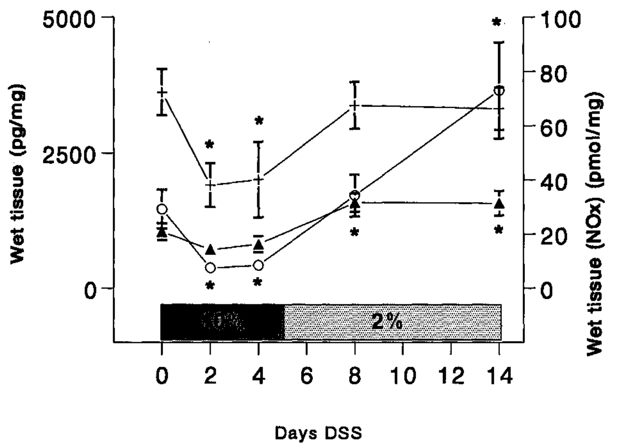

FIG. 3. Production of prostaglandins $6kPGF_{1\alpha}$ (+) and $PGE_2$ (▲) and $NO_2/NO_3$ (○) during the course of DSS-induced colitis. *$p < 0.05$ *vs.* day 0.

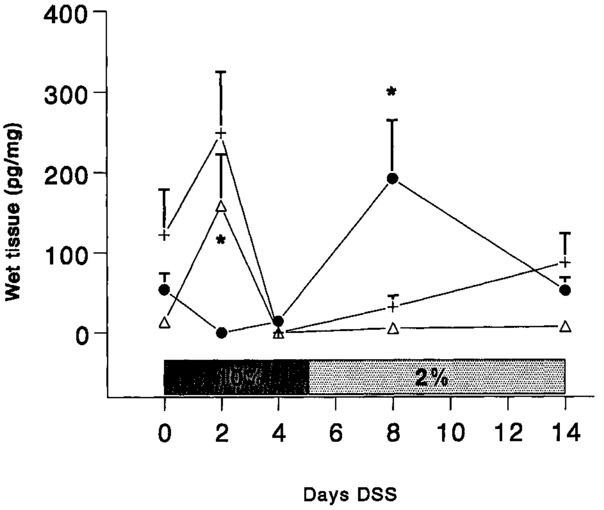

FIG. 4. Production of cytokines TNFα, ((+), units × $10^{-3}$) and IL-1β ((△), units × $10^{-1}$) and PAF (●) during the time-course of DSS-induced colitis. *$p < 0.05$ *vs.* day 0.

DSS concentration in the drinking water was reduced to 2% (w/v) on the fifth day of the treatment schedule, showed a significant decrease of the inflammation score compared with groups which had been treated with 10% DSS for 2 or 4 days. Compared with the control group, however, inflammatory activity was still present (Fig. 2(c)).

*Release of inflammatory mediators:* The mean (± S.E.M.) protein content was, respectively, 11.7 ± 0.7, 4.0 ± 1.0, 9.1 ± 0.8, 9.7 ± 0.4 and 10.9 ± 0.4 mg/g wet tissue on days 0, 2, 4, 8 and 14. The mean (± S.E.M.) MPO activity was, respectively, 106 ± 9.5, 94 ± 22, 60± 13.7, 99± 16.9 and 80 ± 12.0 ng/g wet tissue, resulting in mean concentrations of 9.1, 23.5, 6.6, 10.2 and 7.3 ng MPO/mg protein.

The production of NO, $6kPGF_{1\alpha}$ and $PGE_2$ in 100 mg tissue decreased significantly during the initial phase (d2–d4) of the inflammation (Fig. 3). The production of $LTB_4$ and $LTC_4$ was extremely low in comparison with PGs (1–4 *vs.* 500–3500 pg/mg wet tissue, data not shown) and did not change significantly. The production of the cytokines TNFα and IL-1β was only increased in the first two days (initial phase) of DSS treatment. The production of PAF was unchanged during the initial phase, whereas a marked increase of PAF production was observed (d8) after the concentration of DSS was reduced (Fig. 4).

### Discussion

Dextran sodium sulfate induced a diffuse inflammatory response in the colonic mucosae of the mice when given according to the method described by Okayasu *et al.*[15] A clear correlation between macroscopic and microscopic inflammation scores was observed. Inflammation reached maximal values of approximately 60% of full scale after 4 days treatment with 10% DSS. Further addition of 2% DSS to the drinking water resulted in a degree of chronic inflammation, resembling 25% of the total inflammation score. The individual standard deviations of the groups sacrificed at each time point during the course of the disease are relatively small. Our observations regarding a fast release of pro-inflammatory cytokines confirmed the finding of Rachmilewitz *et al.*[18] that IL-1 was detected in high concentrations during the onset of the inflammation in TNB/ethanol-treated rats. Interleukin-1 was associated with colonic myeloperoxidase activity, representing neutrophil influx. In the TNB/ethanol rat model, the highest $LTB_4$ levels were reached after 1 week, whereas $TxB_2$ was unchanged.[18] In DSS-induced colitis in mice, we now observed a sequential release of cytokines (TNFα and IL-1β) and lipid mediators. The initial release and subsequent effects of pro-inflammatory cytokines have been reviewed recently by Radford-Smith and Jewell.[19] It has been suggested recently that TNFα might stimulate IL-8 production, which in turn activates neutrophils.[20] The secondary release of PAF could result in an increased vascular permeability, which might induce mucus loss and diarrhoea. The formation of the cyclooxygenase products $6kPGF_{1\alpha}$ and $PGE_2$ was significantly decreased during the initial phase of the inflammation. A similar profile was observed for nitric oxide (NO). Both NO and prostacyclin synthase are highly susceptible to attack by free radicals.[21] In our observations, the elevated release of MPO on day 2 indeed precedes the diminished elevation of NO. Endothelial cells could be depleted and unable to generate these bioactive substances. The formation of neutrophil aggregates has been described in PAF-induced gastrointestinal damage,[22] whereas it has been demonstrated that NO can prevent neutrophil adhesion to the endothelium.[23] A significant increase of NO was observed after 8 days of inflammation. This could be due to a secondary influx of activated macrophages which may contribute to this phenomenon. Corticosteroids are known to inhibit the production of both eicosanoids[7] and the pro-inflammatory IL-1$\beta$.[24] These non-selective inhibitory actions could explain why both the initial and the chronic phase of inflammation are successfully treated by this group of compounds. Sulfasalazine derived compounds such as 5-amino-salicylates (5-ASA) non-selectively inhibit eicosanoid formation in dextran sulfate induced colitis in mice,[25] whereas we previously found that 5-ASA increases rather than inhibits PAF synthesis.[16] This effect of 5-ASA on PAF might explain the fact that exacerbations during chronic colitis are well treated by 5-ASA compounds, but total remission is not achieved in many cases.

Further investigations with specific synthesis inhibitors and soluble receptor antagonists are needed to gain more insight in the importance of the sequential release of pro- and anti-inflammatory mediators during acute and chronic colitis.

### References

1. Beeken WL. Experimental inflammatory bowel disease. In: Kirsner JB, Shorter RG, eds. *Inflammatory Bowel Disease, 3rd edition.* Philadelphia: Lea & Febiger, 1988; 37–49.
2. Sharon P, Stenson WF. Metabolism of arachidonic acid in acetic acid colitis in rats. *Gastroenterology* 1985; **88**: 55–63.
3. Schumert R, Towner J, Zipser RD. Role of eicosanoids in human and experimental colitis. *Dig Dis Sci* 1988; **33**: 58S–64S.
4. Fretland DJ, Djuric SW, Gaginella TS. Eicosanoids and inflammatory bowel disease: regulation and prospects for therapy. *Prostagl Leukotr Ess Fatty Acids* 1990; **41**: 215–233.
5. Zipser RD, Nast CC, Lee M, Kao HW, Duke R. *In vivo* production of leukotriene $B_4$ in rabbit colitis. *Gastroenterology* 1987; **92**: 33–39.
6. Laursen LS, Naesdal J, Bukhave K, Lauritsen K, Rask-Madsen J. Selective 5-lipoxygenase inhibition in ulcerative colitis. *Lancet* 1990; **335**: 683–685.
7. Lauritsen K, Laursen LS, Bukhave K, Rask-Madsen J. Effects of topical 5-aminosalicylic acid and prednisolone on prostaglandin $E_2$ and leukotriene $B_4$ levels determined by equilibrium *in vivo* dialysis of rectum in relapsing ulcerative colitis. *Gastroenterology* 1986; **91**: 837–844.
8. Vilaseca J, Salas A, Guarner F, Rodriguez R, Malagelada JR. Participation of thromboxane and other eicosanoid synthesis in the course of experimental inflammatory colitis. *Gastroenterology* 1990; **8**: 269–277.
9. Fedorak RN, Empey LR, MacArthur C, Jewell LD. Misoprostol provides a colonic mucosal protective effect during acetic acid-induced colitis in rats. *Gastroenterology* 1990; **98**: 615–625.
10. Wardle TD, Hall L, Turnberg LA. Inter-relationships between inflammatory mediators released from colonic mucosa in ulcerative colitis and their effect on colonic secretion. *Gut* 1993; **34**: 503–508.
11. Zijlstra FJ, van Dijk APM, Wilson JHP, van Riemsdijk-Overbeeke IC, Vincent JE, Ouwendijk RJT. 15-HETE is the main eicosanoid formed by human colonic mucosa. *Agents and Actions* 1992; **35**: C53–C59.
12. Van Dijk APM, McCafferty DM, Wilson JHP, Zijlstra FJ. 15-Hydroxy-eicosatetraenoic acid has minor anti-inflammatory properties in colitis. *Agents and Actions* 1993; **38**: C120–C121.
13. Morteau O, More J, Pons L, Bueno L. Platelet-activating factor and interleukin 1 are involved in colonic dysmotility in experimental colitis in rats. *Gastroenterology* 1993; **104**: 47–56.
14. Wallace JL. Release of platelet-activating factor (PAF) and accelerated healing induced by a PAF antagonist in an animal model of chronic colitis. *Can J Physiol Pharmacol* 1988; **66**: 422–425.
15. Okayasu I, Hatakeyama S, Yamada M, Ohkusa T, Inagaki Y, Nakaya R. A novel method in the induction of reliable experimental acute and chronic ulcerative colitis in mice. *Gastroenterology* 1990; **98**: 694–702.
16. Phizackerlay PJR, Al-Dabbagh SA. The estimation of nitrate and nitrite in saliva and urine. *Analytical Biochemistry* 1983; **131**: 242–245.
17. Garrelds IM, van Amsterdam JGC, de Graaf-in 't Veld C, Gerth van Wijk R, Zijlstra FJ. Nitric oxide levels in nasal lavages of patients with a house dust mite allergy. *Thorax* 1995; **50**: 275–279.
18. Rachmilewitz D, Simon PL, Schwartz LW, Griswold DE, Fondacaro JD, Wasserman MA. Inflammatory mediators of experimental colitis in rats. *Gastroenterology* 1989; **97**: 326–337.
19. Radford-Smith G, Jewell D.P. The role of cytokines in inflammatory bowel disease. *Mediators of Inflammation* 1994; **3**: 3–9.
20. Raab Y, Gerdin B, Ahlstedt S, Hallgren R. Neutrophil mucosal involvement is accompanied by enhanced local production of interleukin-8 in ulcerative colitis. *Gut* 1993; **34**: 1203–1206.
21. Gryglewski RJ, Palmer RMJ, Moncada S. Superoxide anion is involved in the breakdown of endothelium-derived vascular relaxing factor. *Nature* 1986; **320**: 454–456.
22. Wallace JL, Whittle BJR. Picomole doses of platelet-activating factor predispose the gastric mucosa to damage by topical irritants. *Prostaglandins* 1986; **31**: 989–998.
23. Kubes P, Suzuki M, Granger DN. Nitric oxide: an endogenous modulator of leucocyte adhesion. *Proc Natl Acad Sci USA* 1991; **88**: 4651–4655.
24. Ligumski M, Simon PL, Karmeli F, Rachmilewitz D. Role of interleukin 1 in inflammatory bowel disease — enhanced production during active disease. *Gut* 1990; **31**: 686–689.
25. Zijlstra FJ, Garrelds IM, van Dijk APM, Wilson JHP. Experimental colitis in mice: effects of olsalazine on eicosanoid production in colonic tissue. *Agents and Actions* 1992; **35**: C76–C78.
26. Zijlstra FJ, van Dijk JPM, Wilson JHP. Increased platelet activating factor synthesis in experimental colitis after diclofenac and 5-amino-salicylic acid. *Eur J Pharmacol* 1993; **249**: RI–R2.

ACKNOWLEDGEMENTS. This study was supported by the Metabolica Foundation, Rotterdam. The authors gratefully acknowledge the technical assistance of Ingrid Garrelds, Marjan van Batenburg and Corné Tak.

**Received 13 January 1995; accepted in revised form 7 March 1995**
